# Gains, Losses and Changes of Function after Gene Duplication: Study of the Metallothionein Family

**DOI:** 10.1371/journal.pone.0018487

**Published:** 2011-04-25

**Authors:** Ana Moleirinho, João Carneiro, Rune Matthiesen, Raquel M. Silva, António Amorim, Luísa Azevedo

**Affiliations:** 1 IPATIMUP - Institute of Molecular Pathology and Immunology of the University of Porto, Porto, Portugal; 2 Faculty of Sciences of the University of Porto, Porto, Portugal; Ecole Normale Supérieure de Lyon, France

## Abstract

Metallothioneins (MT) are small proteins involved in heavy metal detoxification and protection against oxidative stress and cancer. The mammalian MT family originated through a series of duplication events which generated four major genes (*MT1* to *MT4*). *MT1* and *MT2* encode for ubiquitous proteins, while *MT3* and *MT4* evolved to accomplish specific roles in brain and epithelium, respectively. Herein, phylogenetic, transcriptional and polymorphic analyses are carried out to expose gains, losses and diversification of functions that characterize the evolutionary history of the MT family. The phylogenetic analyses show that all four major genes originated through a single duplication event prior to the radiation of mammals. Further expansion of the *MT1* gene has occurred in the primate lineage reaching in humans a total of 13 paralogs, five of which are pseudogenes. In humans, the reading frame of all five *MT1* pseudogenes is reconstructed by sequence homology with a functional duplicate revealing that loss of invariant cysteines is the most frequent event accounting for pseudogeneisation. Expression analyses based on EST counts and RT-PCR experiments show that, as for *MT1* and *MT2*, human *MT3* is also ubiquitously expressed while *MT4* transcripts are present in brain, testes, esophagus and mainly in thymus. Polymorphic variation reveals two deleterious mutations (Cys30Tyr and Arg31Trp) in MT4 with frequencies reaching about 30% in African and Asian populations suggesting the gene is inactive in some individuals and physiological compensation for its loss must arise from a functional equivalent. Altogether our findings provide novel data on the evolution and diversification of *MT* gene duplicates, a valuable resource for understanding the vast set of biological processes in which these proteins are involved.

## Introduction

When a particular gene is constrained to a specific function, the appearance of biological novelty demands genetic redundancy. Duplication of pre-existing genes may lead to the establishment of lineage-specific traits and to the development of novel biological functions [1], [2], [3], [4], [5]. However, the probability of widening biological functions (neofunctionalisation) is expectedly lower than the chance of inactivation (pseudogenisation) [6], [7], [8] as most amino-acid replacements are more likely neutral or deleterious, rather than leading to any particular adaptive change. Although the majority of gene duplicates result in pseudogenes, many remain functionally active longer than it would be expected by chance. This observation led to the development of the subfunctionalisation model [9], [10], according to which the accumulation of complementary loss-of-function mutations within regulatory segments of both members would facilitate their preservation while maintaining the original function. In case of preserving the parental function, duplicates may act as backup compensation copies to buffer against the loss of a functionally related gene [11], [12].

The current availability of several genome sequences allows the study of the evolutionary steps underlying the expansion of a gene family by detailed characterisation of lineage-specific expansions. We focused our attention on the mammalian metallothionein family for a number of reasons. MTs are metal-binding proteins involved in homeostasis and the transport of essential metals, more specifically, in protecting cells against heavy metals toxicity [13], [14], having thus a critical role in many biological processes. In mammals, four tandemly clustered genes (*MT1* to *MT4*) are known. Although all genes encode for conserved peptide chains that retain 20 invariant metal-binding cysteines, MT3 and MT4 seem to have developed additional properties relatively to MT1 and MT2, such as protection against brain injuries [15], [16] and epithelial differentiation [17], respectively. Finally, during the evolution of the lineage that led to modern humans, *MT1* has undergone further duplication events that have resulted in 13 younger duplicate isoforms [18]. The co-existence of younger and older duplicates is thus an opportunity to reconstruct the evolutionary history behind the divergence of the MT family in mammals.

## Materials and Methods

### Phylogenetic analyses

Coding sequences annotated as orthologues of the human *MT* genes were extracted from the Ensembl database (www.ensembl.org, release 56: Sep 2009) [19]. The final set of sequences (Table S1) does not include shortened sequences and those annotated in non-human species as representing the orthologue of distinct human *MT1* genes. Codon sequences were aligned using MUSCLE [20], [21] incorporated in Geneious software v5.1.3 (http://www.geneious.com). Coding *MT* sequences from four fish species (*Danio rerio*, *Oryzias latipes*, *Tetraodon nigroviridis* and *Takifugu rubripes*), two birds (*Gallus gallus* and *Taeniopygia guttata*) and a reptile (*Anolis carolinensis*) were used to outgroup the phylogeny. Two methods were used to reconstruct the tree topology: maximum likelihood (ML) and Bayesian. In both cases, the model of nucleotide substitution used was HKY+G as determined in jModelTest [22]. The program BEAST [23] was used to estimate the Bayesian phylogeny in two runs (50 million generations each) using a Bioportal at the University of Oslo (http://www.bioportal.uio.no). The resulting log file was analyzed in Tracer [24]. The tree was obtained in TreeAnnotator from the BEAST software using a threshold for clade credibility of 0.5. For all the statistics obtained, the effective sample size (ESS) was always within the recommended threshold. The ML topology (Figure S1) was obtained with PHYML (http://www.bioportal.uio.no) [25] using the transition/transversion ratio, the proportion of invariable sites and the gamma parameter estimated by the program. Bootstrap branch support was estimated using 1000 data sets. Tree visualization and final edition were performed in FigTree v1.3.1 (http://tree.bio.ed.ac.uk/software/figtree).

### Organization of the human and mouse *MT* family

The chromosomal organization of the *MT* family and flanking neighbours (*BBS2* and *NUP93*) in humans and mice was performed using NCBI (*Homo sapiens* build 36.2 and *Mus musculus* build 37.1) [26] and Ensembl (release 56) genomic coordinates.

### RT-PCR and expressed sequence tag (EST) analyses

Total RNA from 15 human tissues was obtained from Ambion (FirstChoice Human Total RNA Survey Panel). The complementary DNA (cDNA) was synthesized with random hexamer primers from 2 µg of human total RNA using the RETROscrip First Strand Synthesis Kit (Ambion) according to the manufacter's instructions. PCR primers used for amplification of the *MT2*, *MT3* and *MT4* transcripts were: 5′ATCCCAACTGCTCCTGCGCCG3′ (forward) and 5′CAGCAGCTGCACTTGTCCGACG3′ (reverse), 5′CTGAGACCTGCCCCTGCCCTT3′ (forward) and 5′TGCTTCTGCCTCAGCTGCCTCT3′ (reverse) and, 5′CCCCAGGGAATGTGTCTGCATGT3′ (forward) and 5′GGCACATTTGGCACAGCCCGG3′ (reverse), respectively. Samples were amplified with Qiagen Master Mix for 35 cycles at 95°C for 30 sec, 62°C for 30 sec and 72°C for 45 sec after an initial denaturation at 95°C for 15 min and followed by a final extension step of 10 min at 72°C. PCR products were then purified with ExoSAP-IT (USB Corporation, Ohio, USA) by incubation at 37°C for 15 min, followed by enzyme inactivation for 15 min at 85°C. The resulting purified fragments were sequenced using an ABI Big Dye Terminator Cycle Sequencing Ready Reaction kit (Applied Biosystems) and analysed in an ABI PRISM 3130xl (Applied Biosystems) for validation of the corresponding sequence.

ESTs were extracted from Unigene [26] as counts per million transcripts for each of the given tissues and displayed as log_2_ of transcripts per million. A unique EST for human *MT4* is annotated in Unigene (GenBank BF759140.1) but since it contains intronic sequence was removed before plotting the data set. The heat-maps were constructed with an in-house tool using average linkage and the correlation of expression patterns as a measure of dissimilarity to build the hierarchical clusters.

### Polymorphic population data

The Biomart tool [27] at the Ensembl database was used to assess the human polymorphic variability. Allelic and genotypic frequencies of Tyr30 (rs666636) and Trp31 (rs666647) were extracted from the HapMap project (http://www.hapmap.org). Genotypes for each population were also retrieved and analysed to evaluate the linkage disequilibrium between variants.

### Structural homology modelling and visualisation

The crystal structure of the mouse Mt2 [28] (PDB code 4mt2) was used to model [29] both human native and mutation-carrying MT4 proteins following a previously reported methodology [30]. All the structures were displayed with PyMol (www.pymol.org).

## Results

### Evolutionary history of the MT cluster in mammals

**Figure 1:**
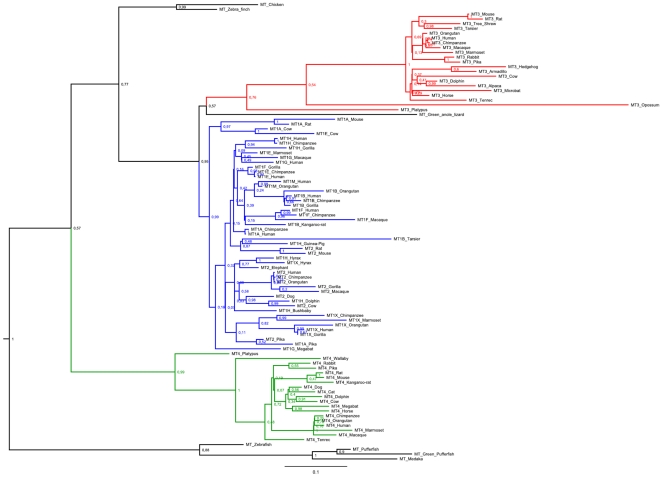
Bayesian phylogenetic analysis of the *MT* family. The gene tree was constructed using coding sequences from the Ensembl database (Table S1). *MT1/MT2*, *MT3* and *MT4* clusters are represented in blue, red and green, respectively. Posterior probability values are given for branch support. Scale bar stands for the number of replacements per site.

The phylogeny of the MT family in mammals was reconstructed with Bayesian and maximum likelihood methods both approaches resulted in a similar topology (Fig. 1 and Figure S1) revealing the robustness of the inference. Tree topology, supported by high values of posterior probabilities (Fig. 1), point clearly to two rounds of duplication occurring at the *MT* family creating *MT4* first and the ancestor of *MT1/MT2/MT3*. In a second round of duplication, *MT3* diverged from the *MT1/MT2* ancestor. These data are in agreement with previous observations [31] which concluded that *MT* duplication occurred before mammalian radiation. However, because *MT* genes from birds and reptiles group within the MT1/*MT2/MT3* cluster, it is possible to consider a more ancient origin for *MT4*, and therefore, its loss in non-mammalian land vertebrates. An alternative explanation is that *MT4* is thus a mammalian-specific gene which has been accumulating a pronounced number of replacements assigning its sequence to a basal position in both Bayesian and ML phylogenies.

**Figure 2:**
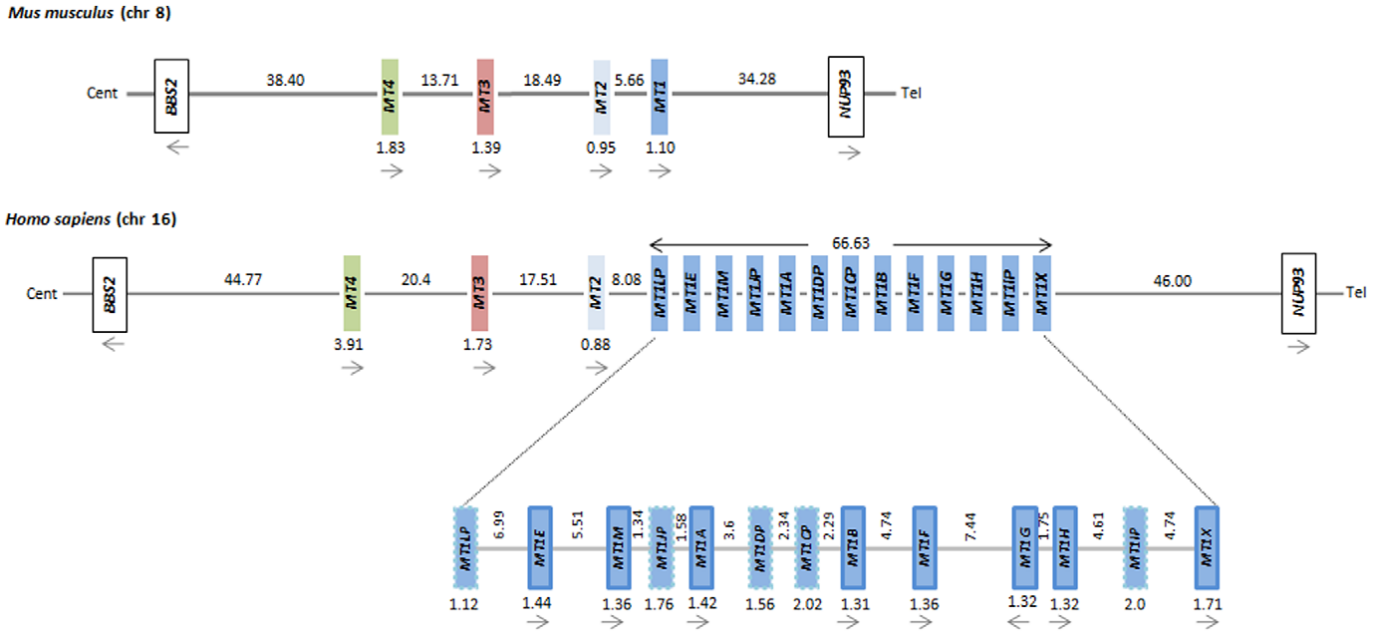
Illustration of the *MT* family in humans and mice. In mice, the family comprises four functionally active genes (*mt1* to *mt4*). In humans, the family harbours a single-copy of *MT2*, *MT3* and *MT4*, and by a tandemly duplicated array of the *MT1* duplicates spanning about 66.6 Kb, where eight active genes (*MT1A* to *MT1J*, *MT1L*, *MT1M* and *MT1X*) and five pseudogenes (*MT1L*, *MT1J*, *MT1D*, *MT1C* and *MT1I*) are known. The direction of transcription for each active gene is indicated by an arrow. Genes are coloured as follows: *MT1* (dark blue), *MT2* (light blue), *MT3* (red) and *MT4* (green). Pseudogenes are represented by dashed boxes. Numbers corresponding to the sizes of gene and intergenic regions are given in Kb.

Extant mammalian genomes seem to carry a single copy of *MT2*, *MT3* and *MT4*, while several *MT1* copies are found in some species. The highest number of *MT1* genes was found in the genome of primates indicating they have arisen in recent duplication events. The detailed genomic organization of the *MT* cluster in humans and mice provides a good example (Fig. 2). In both species, genes are oriented as cent-*MT4-MT3-MT2-MT1*-tel and flanked by *BBS2* and *NUP93*, revealing an evolutionarily conserved arrangement of the cluster. Still, a marked difference distinguishes both genomes. While *mt1* did not expand in mice, humans carry 13 arrayed duplicates (*MT1A* to *MT1J*, *MT1L*, *MT1M* and *MT1X*), five of which (*MT1L*, *MT1J*, *MT1D*, *MT1C* and *MT1I*) have been predicted to be no longer active forms. Overall, this corresponds to a genomic expansion of about 66 Kb in humans.

### Characterisation and divergence of mammalian MT proteins

**Figure 3:**
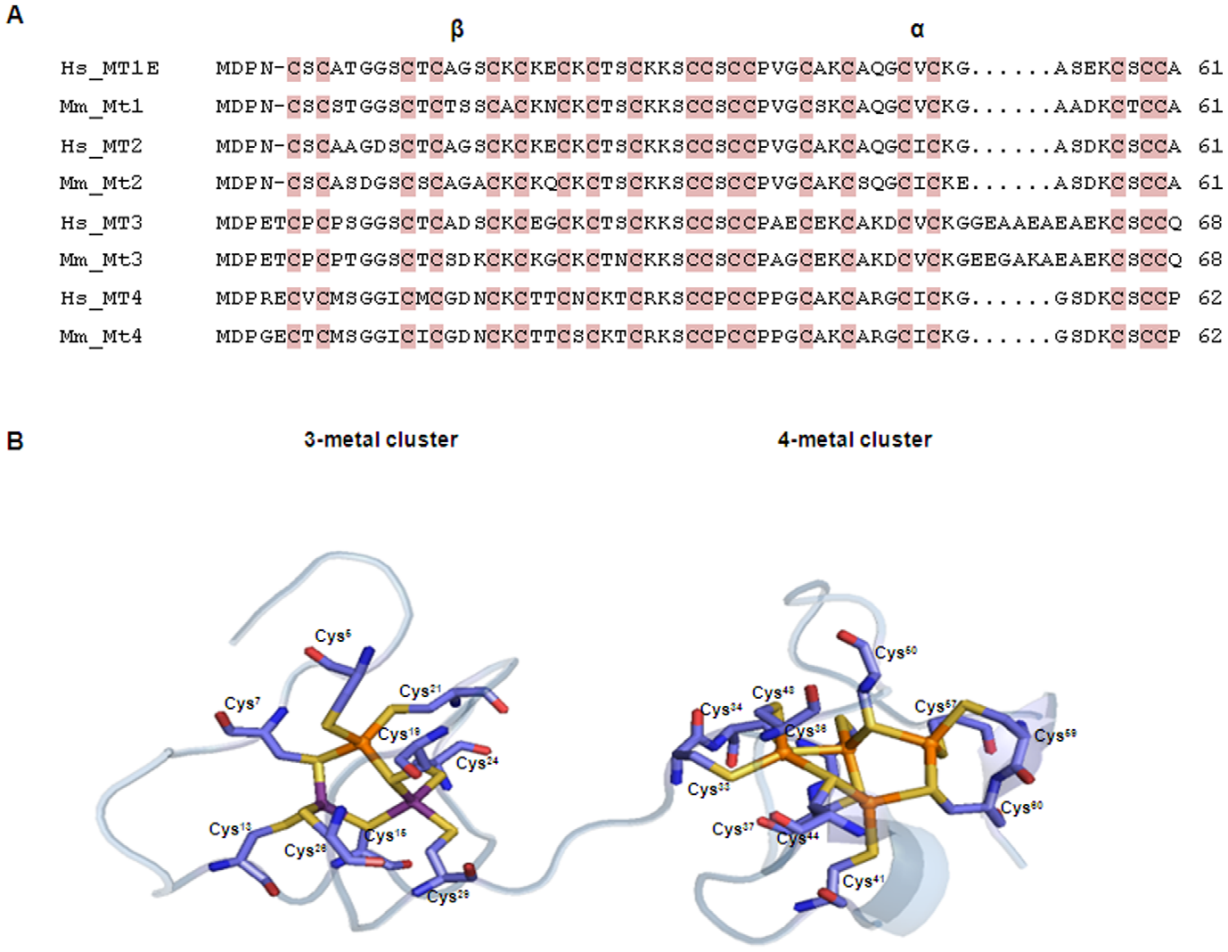
Sequence comparison and structural features of MT proteins. (A) Sequence alignment for human (Hs) and mouse (Mm) proteins. Residues spanning α and β domains are indicated as well as are the invariant metal-binding cysteine residues. Sequences were aligned with ClustalW [59] (B) Structural representation of the mouse Mt2 (PDB 4mt2) showing the two metal-binding clusters and the detailed spatial organisation of the cysteine residues with the S-atoms oriented towards metal ions. Elements are coloured as follows: S (yellow), O (red), N (dark blue), Zn (purple) and Cd (orange).

Mammalian MTs are proteins with 20 invariant cysteines (Fig. 3A) which are responsible for the binding and sequestration of zinc (Zn), cadmium (Cd) and copper (Cu), among other metals. A total of 9 and 11 cysteines are required to form protein-domains that bind three and four ions (Fig. 3B). As documented previously [32], [33] and illustrated here (Fig. 3B), these residues are involved in metal binding through their thiol (-SH) moieties, which must be oriented towards the inside of the metal clusters whenever the ions are sequestered [34]. In humans and mice, MT1 and MT2 are 61-residue proteins (Fig. 3A). The MT3 holds an extra residue in the β domain (Thr5), which is important for its neuroinhibitory activity [35], [36], and a six-residue long insertion in the α domain, whereas MT4 shares an additional residue in the β domain (Glu5) (Fig. 3A).

**Figure 4:**
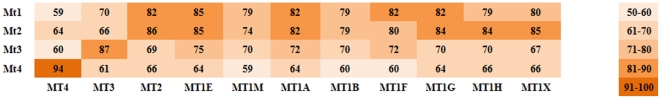
The amino acid identity matrix for human and mouse proteins. Amino acid identity for each pairwise comparison between a human and a mouse protein is given as percentage values.

In order to obtain a clear picture of the amino acid conservation between all pairs of active MTs, identity scores were calculated in human/mouse comparisons (Fig. 4). As shown, MT2, MT3 and MT4 orthologues share 86%, 87% and 94% of residue identity, respectively. Among human MT1 duplicates, MT1E showed the highest identity (85%) with the mouse Mt1. Protein identity scores for MT3 resembles that observed in MT1 and MT2 while MT4 orthologues are strongly conserved in their aminoacid sequences (94% of residue identity between human and mouse). Previously, it was suggested that the preservation of MT4 sequence (only 4 out of 62 residues differed between human and mouse proteins) results from functional constraints [37] involved in epithelial cell differentiation [17]. Phylogenetic analyses (Fig. 1 and Figure S1) show that MT4 resulted from an old event of duplication and the high degree of sequence conservation between human and mouse orthologues strongly points to a role which seems to have been functionally important in mammalian evolution.

### About the pseudogenisation incidents

**Figure 5:**
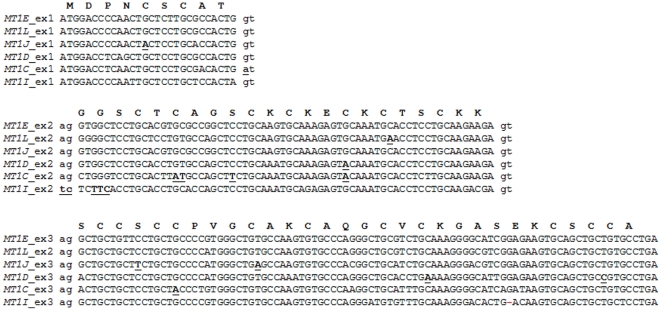
The reconstructed open reading frame of *MT1* pseudogenes. The reconstruction was performed using the genomic sequence of human *MT1* pseudogenes to a homology-based comparison with the functional *MT1E*. Each exon is shown *in a* separate row. Amino-acids are represented in single-letter code above the corresponding codon. Strong deleterious mutation candidates (loss of invariant cysteines, gain of aromatic residues, indels and splice-site mutations) are underlined.

An important aspect related to the fate of a gene copy is the identification of the type of replacements leading to the pseudogenisation of functionally active genes. From its genomic sequence, we would be able to reconstruct the ancestral functional open reading frame and, at the same time, discern the panel of mutational events that have accumulated over time. To reconstruct the open reading frame of all the five *MT1* pseudogenes, the corresponding genomic sequences were aligned with each of the *MT1E* exons separately, followed by manual inspection of sequence homology (Fig. 5). Because any attempt to disclose the impact of nonsynonymous replacements is not straightforward unless complemented with additional functional assays, we focused our attention on obvious damaging mutations, such as (a) replacement of invariant cysteine residues, (b) introduction of aromatic residues, as well as (c) premature stop codons, indels and mutations at the consensus donor (GT) or acceptor (AG) splice sites. Because these are shared features among all functional genes, our inferences are not constrained by the functional template that could have been chosen to infer the reading frame of each pseudogene.

We detected a total of 12 deleterious mutations within the predicted reading frame of *MT1* pseudogenes, eight of which would result in the cysteine replacement and, in some cases, the inclusion of a premature stop codon (Cys5Tyr, Cys15Tyr, Cys24Tyr, Cys26X, Cys37Tyr, Cys41X, Cys50X, and Cys60Arg) covering *MT1JP*, *MT1CP*, *MT1DP* and *MT1LP*, three non-cysteine codons that would encode for an aromatic residue (Gly11Phe, Ser18Phe and Ser35Phe) in *MT1IP* and *MT1CP*, and a 1-bp deletion at the C-terminal domain in *MT1IP*. Since cysteine replacement either with tyrosine or with a stop codon involves only a single nucleotide substitution, these residues are expected to often contribute to MT pseudogeneization.

Outside the coding sequence, two mutations were found at the donor and acceptor splice site of *MT1CP* and *MT1IP*, respectively. Regarding the number of mutations, *MT1CP* is the pseudogene harbouring the highest number of events (six in total), followed by *MT1IP* and *MT1DP* (with three mutations each), *MT1JP* (two mutations) and finally by *MT1LP*, which would encode a truncated protein due to a premature stop codon.

### Expression profile of the *MT* genes in humans and mice

**Figure 6:**
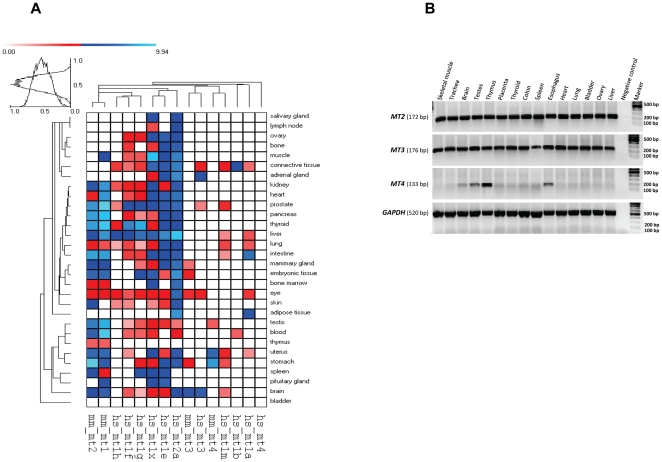
Expression profile of *MT* genes. (A) Expression of the *MT* genes in human and mouse tissues represented as log_2_ of EST counts by colour coding. (B) RT-PCR analysis of human *MT2*, *MT3* and *MT4* in 15 tissues using GAPDH as control.

Since gene duplication often results in a diversified spatiotemporal pattern of expression of duplicate family members [38], [39], [40], [41], [42] we next examined the *MT* transcription pattern using EST data for 30 human and mouse tissues as a metric of basal expression for all functional genes. Previous data have shown that *MT1* and *MT2* are ubiquitously expressed in both species whereas *MT3* and *MT4* present a confined expression in human and mouse brain [43] and in mouse epithelial tissue [17] respectively, although data regarding expression of *MT4* in human tissues is still missing in the literature. The EST records were assembled in a diagram (Fig. 6A) that, in general terms, strongly overlaps the literature data. For instance, mouse *mt1* and *mt2* seem to be as widely expressed as the human *MT2*, a gene that has been proposed to have a housekeeping role for heavy metal homeostasis in every cell [44]. In humans, the expression pattern of *MT2* clusters with *MT1E* and *MT1X*, the two duplicates that reveal the most wide pattern of basal expression [45]. The remaining genes revealed a more confined pattern of basal expression. For instance, constitutive expression of *MT1B* seems to be restricted to connective and blood tissues, whereas *MT1A* is highly expressed in the intestine and adipose tissue and less in uterus, eye, liver and lung. This analysis also rank *MT1H*, *MT1F* and *MT1G* in an intermediate position, showing a pattern that is not as wide as that of *MT1E* and *MT1X* but not so restricted as that of *MTB*, *MT1A* and *MT1M* either. Although the lack of ESTs in particular tissues may indicate difficulties to distinguish between different *MT1* transcripts or bias in tissue representation, these caveats do not necessarily challenge the tissue-specificity generally observed in most *MT1* duplicates, possibly playing distinct roles in distinct cell types as was suggested before [44], [46].

In contrast to *MT1* and *MT2*, both *MT3* and *MT4* are constitutive tissue-specific isoforms that do not respond to metal-induction [17], [44], [47], [48], [49]. The EST data (Fig. 6) although supporting high expression of *MT3* in human and mouse brain tissues also reveal abundant expression in other tissues as well.

Concerning the *MT4* profile, EST records agree with previous findings that documented the stratified squamous epthitelium of digestive and reproductive systems as the main source of gene transcripts in mouse [17]. Thus far, no data exist on the expression of *MT4* in humans (reviewed in [31]) and the EST surveillance has not detected expression in any tissue. These intriguing observations directed a more refined analysis concerning *MT4* expression in humans that was here accomplished by RT-PCR in 15 human tissues. For comparative purposes we also assayed the expression of *MT2* and *MT3* in the same tissue collection (Fig. 6B). As expected, transcripts related with *MT2* were observed in all tissues analysed. A similar scenario was observed for *MT3* which is here demonstrated to be as ubiquitously expressed as is *MT2*. On the other hand, the detection of *MT4* transcripts was only possible in four tissues (brain, testes, thymus and esophagus), with an evident higher expression in thymus, whose medullar component is mainly composed of epithelial cells [50]. Although the RT-PCR itself cannot provide exact quantitative inferences, it is possible to infer that in all the tissues where *MT4* transcripts were detected, the expression level resulted lower than that of *MT2* and *MT3* with the exception of thymus.

### Human polymorphic variability at the MT cluster

Although it is well established that functional MTs preserve a sequence with 20 invariants cysteines, no studies have thus far established the polymorphic status of the remaining residues. To achieve such information, we retrieved the available information on all human active proteins regarding nonsynonymous replacements (Table 1). Several nonsynonymous variants were found in MT1 duplicates and in MT2, but none is documented for MT3. Although a link between any of these replacements and a functional perturbation is not straightforward without complementary experimental assays, it should be mentioned that, in most of the cases the conserved cysteine residues remain unchanged and the replacement does not introduce an aromatic amino acid. The exceptions were observed in MT1B and MT4. In the MT1B case, a putative deleterious Cys19Ser (rs61744104) is indicated as polymorphic in humans although no additional data concerning the allelic frequencies are available in the databases. In the MT4 case, two candidate deleterious mutations were detected, Cys30Tyr (rs666636) and Arg31Trp (rs666647), both of which result in the introduction of aromatic amino acids along with the substitution of a critical cysteine at position 30. Taking into account the functional requisites of MTs, any of these mutations would ultimately result in an impaired metal-binding protein. In contrast with the MT1B case, allelic frequencies for these two polymorphisms are available and were retrieved from the HapMap (Table 2). Allelic frequencies of Cys30Tyr (rs666636) and Arg31Trp (rs666647) in 11 populations are presented in Table 2. In European populations up to 8,5% of the individuals carry a putatively deleterious allele. Frequencies are even higher in African and Asian populations (up to 30%). Genotype evaluation in all the populations showed that every chromosome that contains the Trp31 allele also contains the Tyr30, but not vice versa, which points to Cys30Tyr as the oldest variant and Arg31Trp as appearing afterwards in the same background.

**Table 1:** Nonsynonymous replacements at the human *MT* genes annotated in Ensembl database.

Gene	Nonsynonymous variants
*MT1E*	Asn40Ser (rs12051120)
	Arg46Lys (rs34166523)
*MT1M*	Thr20Lys (rs1827210)
*MT1A*	Thr27Asn (rs11640851)
	Lys51Arg (rs8052394)
*MT1B*	Cys19Ser (rs61744104)
*MT1F*	-
*MT1G*	-
*MT1H*	Gly17Arg (rs9934181)
*MT1X*	Lys22Asn (rs17851637)
*MT2*	Ala42Val (rs35109646)
*MT3*	-
*MT4*	Cys30Tyr (rs666636)
	Arg31Trp (rs666647)
	Gly48Asp (rs11643815)

**Table 2:** Hapmap allelic frequencies of *MT4* Tyr30 (rs666636) and Trp31 (rs666647) in human population.

Population	TYR^30^-A allele	TRP^31^-T allele
**African**		
ASW	0.189	0.189
LWK	0.303	0.244
MKK	0.155	0.150
YRI	0.161	0.124
**Asian**		
CHB	0.298	0.298
CHD	0.288	0.282
JPT	0.267	0.265
GIH	0.045	0.045
**European**		
CEU	0.085	0.058
TSI	0.034	0.034
**American**		
MEX	0.05	0.05

Population description as indicated in Hapmap: ASW, African ancestry in Southwest USA; CEU, Utah residents with Northern and Western European ancestry from the CEPH collection; CHB, Han Chinese in Beijing, China; CHD, Chinese in Metropolitan Denver, Colorado; GIH, Gujarati Indians in Houston, Texas; JPT, Japanese in Tokyo, Japan; LWK, Luhya in Webuye, Kenya; MEX, Mexican ancestry in Los Angeles, California; MKK, Maasai in Kinyawa, Kenya; TSI, Toscans in Italy; and YRI, Yoruba in Ibadan, Nigeria.

## Discussion

The history and fate of post-duplication events in the mammalian evolution was herein explored by the study of MT family members. After a duplication event, newly arisen genes can follow distinct evolutionary paths: if the parental gene is maintained active, redundant duplicates can escape purifying selection and start to accumulate loss-of-function mutations resulting in pseudogeneisation; less frequently, particular replacements may direct new genes into novel functions. Subfunctionalisation can also occur if parental and duplicates retain function but become distinct and complementary in their spatiotemporal pattern of expression.

Mammalian MT1 and MT2 are conserved proteins that play a critical role in heavy-metal homeostasis and are transcriptionally induced by metal [51] and glucocorticoids [52]. While most of the mammals show a single *MT1* and *MT2* copy that evolved through a duplication event, primates harbor multiple *MT1* copies (Fig. 1). In humans, *MT1* expansion resulted in a total of 13 tandemly arranged genes, five of which are known or predicted to be pseudogenes, while the remaining eight are still functionally active (Fig. 2). The pseudogeneization process has occurred by the accumulation of loss-of-function mutations mainly by replacing critical metal-binding cysteines or incorporated aromatic amino-acids in the protein sequence (Fig. 5). The most recently documented of these pseudogenes, *MT1L* [53], shows a unique mutation in which an invariant cysteine is replaced by a premature stop codon (Cys26Stop) resulting in a truncated protein.

Since its discovery [54], MT3 has been frequently associated with the protection against neuronal injury [15], [16]. The mammalian MT3 protein shows a characteristic insertion of six residues at the α-domain when compared to that of MT1 and MT2 (Fig. 3) and an extra residue in the β domain (Thr), which is responsible for neuron growth inhibitory activity in Alzheimer disease [35], [36]. Although the expression of *MT3* has been almost exclusively related to brain tissues, we demonstrate that *MT3* is a ubiquitously expressed gene. These results would drive future investigations on the involvement of MT3 in other cellular processes. In this regard, it is worth mentioning that Mt3 associates with other proteins in mouse brains as part of a multiprotein complex [55] suggesting function diversification and involvement in various physiological processes.

The most recently discovered family member, MT4, retains a high degree of conservation between humans and mice (Fig. 4), yet it shows the highest sequence divergence when compared with any other MT family member. However, the detection of structurally disrupting mutations at polymorphic proportions (Table 1) predicts that MT4 is inactive in some individuals. If that is the case, might the role of MT4 be performed by another family member? To address this possibility, the metal binding properties of MT4 were explored in the literature data. It has been shown that mouse Mt4 retains the capacity to bind Zn [17], [37], Cd and Cu as the ubiquitously expressed Mt1/Mt2, although the affinity to Cu is higher [37], [56]. Furthermore, it showed similar characteristic metal-thiolate clusters and solvent accessibility as Mt1 [57]. Extrapolating these properties to the human protein, for which no data of such detail are available, it is tempting to assume that the loss (pseudogeneization) of *MT4* can be compensated by functional equivalents. In this context, *MT1* and *MT2* would be the most likely candidates for a number of reasons. First, the metal binding properties of Mt1 and Mt2 overlap that of Mt4 in mice [57]. Second, it has been demonstrated that some *MT1* duplicates have cellular specificity [44], [46] and some of them are expressed in epithelium. Third, previous experiments in *Drosophila melanogaster* demonstrated that the number of functional gene duplications correlates to the resistance to Cd [13], [58] and Cu [13] as a direct consequence of the increased gene expression. Taken these data together, is thus possible that additional *MT1* duplicates may have assumed the specialized role of the inactivated protein in humans. In such a case, it is likely that some *MT1* genes may act as compensatory backup copies that replace *MT4* in individuals carrying deleterious mutations. Accordingly, it is thus further possible to infer that the compensatory mechanism implies the regulation of gene expression as observed in *D. melanogaster*.

Tracing the evolutionary history of a gene after duplication often leaves more questions than answers. Some of those answers are easily obtained by direct read of DNA or protein sequences while some other depend on additional information which was here gathered for the *MT* family. Data on phylogeny, expression and intra-specific polymorphic information are necessary to drive hypotheses regarding the gain, loss and change of function of duplicated genes. The application of such a network of information, as is the case of this study, is thus necessary to extend the knowledge about the evolutionary fate of genes originated by duplication events.

## Supporting Information

Figure S1 Maximum likelihood analysis of the MT family. The gene tree was constructed using coding sequences from the Ensembl database (Table S1). *MT1*/*MT2*, *MT3* and *MT4* clusters are represented in blue, red and green, respectively. Branch support was estimated by bootstrap. Scale bar: number of replacements per site.
(TIF)

Table S1 Metallothionein gene transcripts annotated in the Ensembl database (release 56, Sep 2009) as human orthologues of *MT1E*, *MT1M*, *MT1A*, *MT1B*, *MT1F*, *MT1G*, *MT1H*, *MT1X*, *MT2*, *MT3* and *MT4* in mammals. *MT* sequences from fishes, birds and reptiles are shown at the bottom of the table.
(DOC)
