# Supplementary figures and images for: Gains, Losses and Changes of Function after Gene Duplication: Study of the Metallothionein Family
Source: PLoS One. 2011 Apr 25;6(4):e18487. doi: 10.1371/journal.pone.0018487 (PMC3081807; doi:10.1371/journal.pone.0018487)

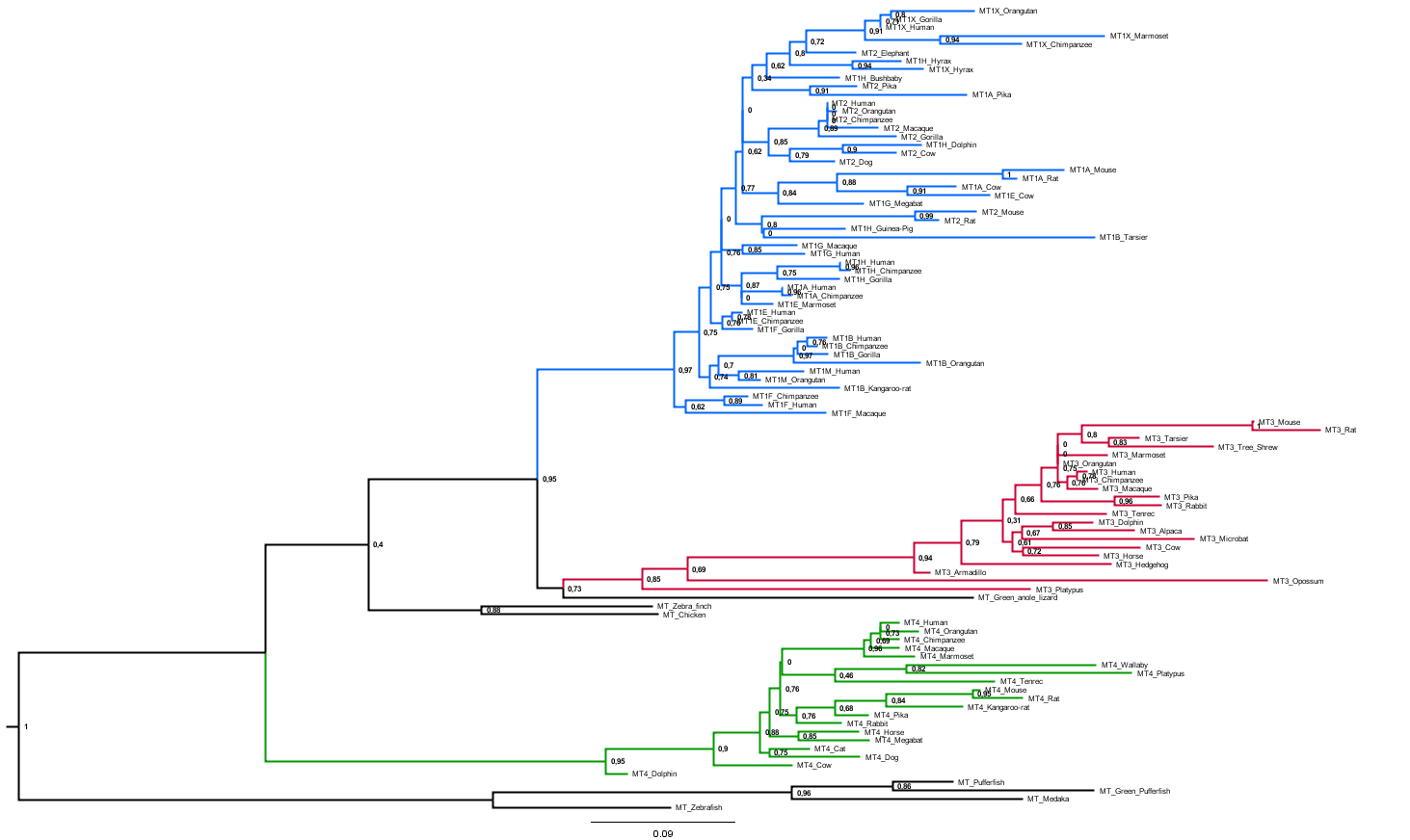

Supplement: Figure S1 — Maximum likelihood analysis of the MT family. The gene tree was constructed using coding sequences from the Ensembl database (Table S1). MT1/MT2, MT3 and MT4 clusters are represented in blue, red and green, respectively. Branch support was estimated by bootstrap. Scale bar: number of replacements per site. (TIF) [file pone.0018487.s001.tif]
