# Supplementary material for: Gains, Losses and Changes of Function after Gene Duplication: Study of the Metallothionein Family
Source: PLoS One. 2011 Apr 25;6(4):e18487. doi: 10.1371/journal.pone.0018487 (PMC3081807; doi:10.1371/journal.pone.0018487)
Supplement: Table S1 — Metallothionein gene transcripts annotated in the Ensembl database (release 56, Sep 2009) as human orthologues of MT1E, MT1M, MT1A, MT1B, MT1F, MT1G, MT1H, MT1X, MT2, MT3 and MT4 in mammals. MT sequences from fishes, birds and reptiles are shown at the bottom of the table. (DOC) [file pone.0018487.s002.doc]

**Table S1.** Metallothionein gene transcripts annotated in the Ensembl database (release 56, Sep 2009) as human orthologues of *MT1E*, *MT1M*, *MT1A*, *MT1B*, *MT1F*, *MT1G*, *MT1H*, *MT1X*, *MT2*, *MT3* and *MT4* in mammals. *MT* sequences from fishes, birds and reptiles are shown at the bottom of the table.

| **Gene** | **Scientific name** | **Common name** | **Ensembl transcript** |
| --- | --- | --- | --- |
| ***MT1E*** | *Bos taurus* | Cow | ENSBTAT00000046456 |
|  | *Pan troglodytes* | Chimpanzee | ENSPTRT00000063817 |
|  | *Homo sapiens* | Human | ENST00000306061 |
|  | *Callithrix jacchus* | Marmoset | ENSCJAT00000024529 |
|  |  |  |  |
| ***MT1M*** | *Homo sapiens* | Human | ENST00000379818 |
|  | *Pongo pygmaeus* | Orangutan | ENSPPYT00000029425 |
|  |  |  |  |
| ***MT1A*** | *Bos taurus* | Cow | ENSBTAT00000002092 |
|  | *Ochotona princeps* | Pika | ENSOPRT00000006730 |
|  | *Pan troglodytes* | Chimpanzee | ENSPTRT00000015005 |
|  | *Homo sapiens* | Human | ENST00000290705 |
|  | *Mus musculus* | Mouse | ENSMUST00000034215 |
|  | *Rattus norvegicus* | Rat | ENSRNOT00000038212 |
|  |  |  |  |
| ***MT1B*** | *Pan troglodytes* | Chimpanzee | ENSPTRT00000015003 |
|  | *Gorilla gorilla* | Gorilla | ENSGGOT00000009888 |
|  | *Homo sapiens* | Human | ENST00000334346 |
|  | *Pongo pygmaeus* | Orangutan | ENSPPYT00000009031 |
|  | *Tarsius syrichta* | Tarsier | ENSTSYT00000007085 |
|  | *Dipodomys ordii* | Kangaroo rat | ENSDORT00000003898 |
|  |  |  |  |
| ***MT1F*** | *Pan troglodytes* | Chimpanzee | ENSPTRT00000015004 |
|  | *Gorilla gorilla* | Gorilla | ENSGGOT00000009872 |
|  | *Homo sapiens* | Human | ENST00000334350 |
|  | *Macaca mulatta* | Macaque | ENSMMUT00000039321 |
|  |  |  |  |
| ***MT1G*** | *Pteropus vampyrus* | Megabat | ENSPVAT00000016198 |
|  | *Homo sapiens* | Human | ENST00000379811 |
|  | *Macaca mulatta* | Macaque | ENSMMUT00000022653 |
|  |  |  |  |
| ***MT1H*** | *Tursiops truncatus* | Dolphin | ENSTTRT00000013550 |
|  | *Procavia capensis* | Hyrax | ENSPCAT00000013294 |
|  | *Otolemur garnettii* | Bushbaby | ENSOGAT00000008589 |
|  | *Pan troglodytes* | Chimpanzee | ENSPTRT00000014999 |
|  | *Gorilla gorilla* | Gorilla | ENSGGOT00000016717 |
|  | *Homo sapiens* | Human | ENST00000332374 |
|  | *Cavia porcellus* | Guinea Pig | ENSCPOT00000011224 |
|  |  |  |  |
| ***MT1X*** | *Procavia capensis* | Hyrax | ENSPCAT00000007828 |
|  | *Pan troglodytes* | Chimpanzee | ENSPTRT00000067756 |
|  | *Gorilla gorilla* | Gorilla | ENSGGOT00000015825 |
|  | *Homo sapiens* | Human | ENST00000394485 |
|  | *Callithrix jacchus* | Marmoset | ENSCJAT00000023761 |
|  | *Pongo pygmaeus* | Orangutan | ENSPPYT00000002180 |
|  |  |  |  |
| ***MT2*** | *Bos taurus* | Cow | ENSBTAT00000034373 |
|  | *Canis familiaris* | Dog | ENSCAFT00000014487 |
|  | *Ochotona princeps* | Pika | ENSOPRT00000001091 |
|  | *Pan troglodytes* | Chimpanzee | ENSPTRT00000014994 |
|  | *Gorilla gorilla* | Gorilla | ENSGGOT00000012065 |
|  | *Homo sapiens* | Human | ENST00000245185 |
|  | *Macaca mulatta* | Macaque | ENSMMUT00000031285 |
|  | *Pongo pygmaeus* | Orangutan | ENSPPYT00000008667 |
|  | *Loxodonta africana* | Elephant | ENSLAFT00000012833 |
|  | *Mus musculus* | Mouse | ENSMUST00000034214 |
|  | *Rattus norvegicus* | Rat | ENSRNOT00000067391 |
|  |  |  |  |
| ***MT3*** | *Vicugna pacos* | Alpaca | ENSVPAT00000003704 |
|  | *Bos taurus* | Cow | ENSBTAT00000022460 |
|  | *Echinops telfairi* | Lesser hedgehog tenrec | ENSETET00000000091 |
|  | *Tursiops truncatus* | Dolphin | ENSTTRG00000013549 |
|  | *Myotis lucifugus* | Microbat | ENSMLUT00000005003 |
|  | *Dasypus novemcinctus* | Armadillo | ENSDNOT00000015796 |
|  | *Erinaceus europaeus* | Hedgehog | ENSEEUT00000003639 |
|  | *Ochotona princeps* | Pika | ENSOPRT00000006724 |
|  | *Oryctolagus cuniculus* | Rabbit | ENSOCUT00000016238 |
|  | *Equus caballus* | Horse | ENSECAT00000015904 |
|  | *Pan troglodytes* | Chimpanzee | ENSPTRT00000014993 |
|  | *Homo sapiens* | Human | ENST00000200691 |
|  | *Macaca mulatta* | Macaque | ENSMMUT00000010829 |
|  | *Callithrix jacchus* | *Marmoset* | ENSCJAT00000024548 |
|  | *Pongo pygmaeus* | Orangutan | ENSPPYT00000008666 |
|  | *Tarsius syrichta* | Tarsier | ENSTSYT00000001126 |
|  | *Mus musculus* | Mouse | ENSMUST00000034211 |
|  | *Rattus norvegicus* | Rat | ENSRNOT00000025669 |
|  | *Tupaia belangeri* | Tree Shrew | ENSTBET00000016034 |
|  | *Monodelphis domestica* | Opossum | ENSMODT00000040198 |
|  | *Ornithorhynchus anatinus* | Platypus | ENSOANT00000029863 |
|  |  |  |  |
| ***MT4*** | *Bos taurus* | Cow | ENSBTAT00000020072 |
|  | *Echinops telfairi* | Lesser hedgehog tenrec | ENSETET00000005977 |
|  | *Felis catus* | Cat | ENSFCAT00000008674 |
|  | *Canis familiaris* | Dog | ENSCAFT00000014504 |
|  | *Tursiops truncatus* | Dolphin | ENSTTRT00000013548 |
|  | *Pteropus vampyrus* | Megabat | ENSPVAT00000014588 |
|  | *Macropus eugenii* | Wallaby | ENSMEUT00000011205 |
|  | *Ochotona princeps* | Pika | ENSOPRT00000006718 |
|  | *Oryctolagus cuniculus* | Rabbit | ENSOCUT00000017267 |
|  | *Equus caballus* | Horse | ENSECAT00000014386 |
|  | *Pan troglodytes* | Chimpanzee | ENSPTRT00000014992 |
|  | *Homo sapiens* | Human | ENST00000219162 |
|  | *Macaca mulatta* | Macaque | ENSMMUT00000022652 |
|  | *Callithrix jacchus* | *Marmoset* | ENSCJAT00000024551 |
|  | *Pongo pygmaeus* | Orangutan | ENSPPYT00000008665 |
|  | *Dipodomys ordii* | Kangaroo rat | ENSDORT00000015428 |
|  | *Mus musculus* | Mouse | ENSMUST00000034207 |
|  | *Rattus norvegicus* | Rat | ENSRNOT00000025694 |
|  | *Ornithorhynchus anatinus* | Platypus | ENSOANT00000029863 |

**Fishes**

| *Danio rerio* | Zebrafish | ENSDART00000061007 |
| --- | --- | --- |
| *Oryzias latipes* | Medaka | ENSORLT00000019509 |
| *Tetraodon nigroviridis* | Green putterfish | ENSTNIT00000011862 |
| *Takifugu rubripes* | Putterfish | ENSTRUT00000022487 |

**Bird**s

| *Taeniopygia guttata* | Zebra finch | ENSTGUT00000006787 |
| --- | --- | --- |
| *Gallus gallus* | Chicken | ENSGALT00000023565 |

**Reptiles**

| *Anolis carolinensis* | Anole lizard | ENSACAT00000007496 |
| --- | --- | --- |
